# The utility of PET/CT in large vessel vasculitis

**DOI:** 10.1038/s41598-020-73818-2

**Published:** 2020-10-19

**Authors:** Jennifer Ben Shimol, Howard Amital, Merav Lidar, Liran Domachevsky, Yehuda Shoenfeld, Tima Davidson

**Affiliations:** 1 Department of Medicine, E. Wolfson Medical Center, Holon, Israel; 2 Department of Medicine, ‘B’ and Zabludowicz Center for Autoimmune Diseases, Chaim Sheba Medical Center, Tel Hashomer, Israel; 3 Center for Autoimmune Diseases, Chaim Sheba Medical Center, Tel Hashomer, Israel; 4 Department of Nuclear Medicine, Chaim Sheba Medical Center, Tel Hashomer, Israel; 5 Sackler Faculty of Medicine, Tel Aviv University, Tel Aviv, Israel; 6 Saint Petersburg Research Institute of Phthisiopulmonology, Saint-Petersburg, Russian Federation

**Keywords:** Rheumatic diseases, Vasculitis syndromes

## Abstract

^18^F-FDG PET/CT occupies a growing role in the diagnosis of large vessel vasculitis (LVV), illustrating enhanced uptake in the lining of large vessels. A retrospective single center study was conducted of patients who underwent ^18^F-FDG PET/CT scans between 2009 and 2019 at Sheba Medical Center, Israel. The imaging results were analyzed for evidence of LVV. We reviewed the PET/CT scans of 126 patients and identified 57 studies that either showed evidence of active LVV or that had been performed in patients previously treated for systemic vasculitis. In 6 patients with fevers of unknown origin and elevated inflammatory markers, PET/CT revealed LVV. Six of 13 patients previously treated for systemic vasculitis demonstrated persistent large vessel uptake. LVV was identified in 8 patients with other autoimmune diseases, and in 4 diagnosed with infectious aortitis. In 26 patients who underwent malignancy surveillance, PET/CT revealed more localized large vessel wall inflammation. Our results illustrate that PET/CT may identify large vessel wall inflammation in patients with a suspicion of LVV, and incidentally in patients who undergo malignancy surveillance. PET/CT may also help delineate the presence and extent of vessel inflammation in patients with LVV and in those with other autoimmune diseases.

## Introduction

18-fluorodeoxy-glucose positron emission tomography/computed tomography (^18^F-FDG PET/CT) has been a mainstay of malignancy surveillance for many years^1^. In the last twenty years, PET/CT has become increasingly recognized as an important tool for rheumatologists in the assessment of large vessel vasculitis (LVV)^2,3^. Uptake of ^18^F-FDG by metabolically active cells helps localize inflammation along the arterial wall where macrophages and other inflammatory cells migrate and reside. In addition to demarcating the location of vessel wall inflammation, PET/CT enables distinguishing vasculitis from atherosclerotic lesions^4^. A typical pattern of more intense inflammation, a continuous stretch of vessel involvement and an absence of calcifications help distinguish vasculitis from the patchier appearance that is typical of atherosclerosis^5^.

For untreated patients with elevated inflammatory markers, the sensitivity of PET/CT in identifying findings consistent with LVV has been reported to range from 77 to 92%, and the specificity from 89 to 100%^6^. In giant cell arteritis (GCA) in particular, most PET/CT's in clinical use are not suitable for evaluating inflammation in the temporal arteries due to their small size and their obscured location. Only using advanced FDG-PET scintigraphy can the temporal, maxillary and occipital be accurately assessed^7^. However, PET/CT remains a useful tool in showing increased tracer uptake in the aorta and in its large proximal branches that are involved in GCA, in up to 45% of patients^8^. PET/CT has also demonstrated considerable utility in outlining disease extent and monitoring disease activity in patients with active Takayasu arteritis, with a sensitivity of 93% and a specificity of 92%^9^.

As clinicians increasingly rely on PET/CT in the assessment of GCA, this imaging modality has shown particular value in patients with non-diagnostic temporal biopsies and in patients who are younger and present with atypical symptoms, such as fever of unknown origin (FUO). In this subset, patients more commonly present with vasculitis involving the extratemporal proximal vessels^10^. Due to its helpfulness, PET/CT has been strongly recommended as a tool for the early diagnosis of vasculitis by both the American College of Rheumatology (ACR) and the European League Against Rheumatism (EULAR)^11,12^. Findings of LVV on PET/CT have been included as possible diagnostic criteria for Takayasu arteritis, in the most recently proposed ACR guidelines^11^.

Despite the clear role of PET/CT in outlining the presence of LVV, its usefulness in monitoring disease activity is less certain. Following treatment with glucocorticoids, the correlation between enhanced vessel uptake and other signs of active inflammation is often mixed^13^. Moreover, the ability of LVV on PET/CT to predict future relapse is inconclusive^14^.

Prior reports do not show a direct association between malignancy and LVV. A recent study that evaluated a large group of patients with well described GCA did not find an increased association with malignancy^15^. Similarly, large cohorts of patients with Takayasu arteritis have not shown evidence of a heightened risk of malignancy^16,17^. Nonetheless, in studies of patients with a history of cancer, PET/CT illustrated enhanced uptake in the walls of the aorta, the subclavian arteries, and the iliofemoral arteries^18,19^. PET/CT cannot distinguish between these lesions and those associated with clinical LVV. Moreover, because 18F-FDG accumulates in regions of the vessel walls where macrophage-rich deposition presents, it cannot discriminate between sterile inflammation and infectious inflammation^4^. PET/CT findings must be interpreted within the context of other clinical information to determine whether findings of enhanced uptake along the arterial wall are consistent with LVV.

The objective of this study was to assess the clinical utility of PET/CT in the diagnosis and monitoring of LVV. Our goal was to characterize the areas of vasculitis, as well as associated imaging features in different subsets of patients. Furthermore, we sought to analyze whether PET/CT is helpful in detecting evidence of disease activity in patients with a history of previously treated vasculitis.

## Materials and methods

### Study design

We searched the computerized database of Sheba Medical Center, a tertiary hospital, for ^18^F-FDG-PET/CT studies that included the term "vasculitis" in their reports from January 2009 through November 2019. The inclusion criterion was evidence of LVV on an ^18^F-FDG PET/CT scan; or a history of systemic vasculitis, regardless of the findings on ^18^F-FDG PET/CT. Imaging data were obtained from the picture archive and communication system (PACS, Carestream Health 11.0, Rochester, NY) and clinical data from the computerized medical records at our hospital. Clinical data including medical history, measurements of inflammatory markers and biopsy results were reviewed. Final diagnoses of GCA and Takayasu arteritis were attributed to those who met ACR 1990 criteria based on clinical and histologic features^20,21^.

### Image assessment

All available images were interpreted by experienced specialists in nuclear medicine and radiology, and re-reviewed by one of the study co-authors, who has dual certification in radiology and nuclear medicine with 20 years’ experience (TD). FDG uptake in the lesions was measured by standardized uptake values max (SUVmax), which were calculated by manually generating a region of interest over the sites of abnormally increased FDG activity^22–24^. Vasculitis was defined by identifying PET areas of increased uptake along the vessels. Abnormal FDG uptake was defined as focal or diffuse uptake higher than the physiological uptake in the liver or mediastinal vascular vessels, and higher than the activity in the surrounding tissue. The distribution of vessel involvement was evaluated on both longitudinal and cross-sectional imaging. In areas of increased vessel wall uptake, CT without contrast was used to assess vessel wall thickening and surrounding fat changes to corroborate the presence of vasculitis. CT images were examined for other findings including in soft tissues, pulmonary opacities and septic emboli that were consistent with inflammatory changes.

### FDG-PET/CT imaging technique

A combined FDG-PET/CT scanner (Philips Gemini GXL, Philips Medical Systems, Cleveland OH, USA) was used, which includes a 16-detector row helical CT. This scanner enables simultaneous acquisition of up to 45 transaxial PET images, with inter-slice spacing of 4 mm in one bed position; and provides an image from the vertex to the thigh with about 10 bed positions. The transaxial field of view and pixel size of the PET images reconstructed for fusion were 57.6 cm and 4 mm, respectively, with a matrix size of 144 × 144. The technical parameters used for CT imaging were: pitch 0.8, gantry rotation speed 0.5 s/rotation, 120 kVp, 250 mAs, 3 mm slice thickness, and specific breath-holding instructions^22–24^.

After 4–6 h of fasting, patients received an intravenous injection of 370 MBq F-18 FDG. About 75 min later, CT images were obtained from the vertex to the mid-thigh for about 32 s. When intravenous contrast material was used, CT scans were obtained 60 s after injection of 2 mL/kg of non-ionic contrast material (Omnipaque 300; GE Healthcare). An emission PET scan followed in 3D acquisition mode for the same longitudinal coverage, 1.5 min per bed position. CT images were fused with the PET data and were used to generate a map for attenuation correction. PET images were reconstructed using a line of response protocol with CT attenuation correction, and the reconstructed images were generated for review on a computer workstation (Extended Brilliance Workstation, Philips Medical Systems, Cleveland OH, USA)^22–24^.

### Clinical management

Patients in whom evidence of LVV was detected incidentally during PET/CT scans for malignancy surveillance were referred for rheumatologic care. Based on the clinical judgment of consulting rheumatologists, decisions regarding further evaluation and treatment were made in the context of multi-disciplinary tumor board conferences, and patients were advised accordingly. In patients with a background of LVV, including GCA or Takayasu arteritis; and in those with other autoimmune diseases not generally associated with aortitis, further work-up and choice of treatment based on the PET/CT findings were decided on a case by case basis, considering all the clinically relevant information available. For all the patients with evidence of LVV, continued monitoring of the vasculitis was advised.

### Ethics

This single-institution study was approved by the institutional review board of Sheba Medical Center, according to the Declaration of Helsinki (approval no: SMC-19–6596). Informed consent was waived by the institutional review board of Sheba Medical Center due to the retrospective nature of the study.

## Results

### Patient characteristics

#### Baseline features

We reviewed the ^18^F-FDG-PET/CT results of 126 patients. A total of 57 patients met the study inclusion criteria. Sixty-nine patients were excluded from the analysis because they did not have a history of a systemic vasculitis and there was no evidence of LVV on their ^18^F-FDG PET/CT scans. Among those excluded, 13 had presented with FUO and had undergone PET/CT scans to evaluate the presence of vasculitis. Of those included, 31 were females. The mean age of the included patients was 59 ± 14.7 years (range 11–82). Eighteen patients had a preexisting history of vasculitis: 10 with known Takayasu arteritis, 3 with GCA, 3 with granulomatosis with polyangiitis (GPA), 1 with primary CNS vasculitis (PCNSV), and 1 with Behçet's disease. One had a preexisting history of hypereosinophilic syndrome (HES), 1 had IgG4-related disease, and 1 had a known history of rheumatoid arthritis (RA) and psoriasis (PsO).

Twenty-six patients had been treated for malignancy. Of them, 20 had a history of a solid tumor. These included melanoma, small cell lung cancer, non-small cell lung cancer, oropharyngeal cancer, breast cancer, stomach cancer, cervical cancer, endometrial cancer, laryngeal cancer, colon cancer, ovarian cancer, and an adrenal tumor. Six patients had a prior hematologic malignancy. The hematologic cancers included diffuse large B cell lymphoma, low grade lymphoma, follicular lymphoma, Hodgkin's lymphoma, and chronic myeloid leukemia (Table 1).

**Table 1:** Indications, demographics, and diagnoses of the cohort.

Indication	Number of studies	Male/female	Age range (mean)	Vascular diagnosis (number of cases)	Non-vascular diagnosis (number of cases)
Assessment of disease activity or vessel involvement in patients with known vasculitis	19	8/11	27–78 (53)	TAK (10) GCA (3) GPA (3) Behçet’s (1) IgG4 disease (1) PCNSV (1)	
Evaluation of suspected vasculitis (for FUO and/or persistently unexplained elevated inflammatory markers)	12	6/6	11–76 (57)	GCA (2) TAK (1) Inflammatory aortitis (3) Infectious aortitis (3) Mycotic aneurysm (1)	HES (1) RA and PsO (1)
Surveillance of known malignancy	26	12/14	44–82 (64)	Incidental vasculitis (26)	Adrenal tumor (1) Breast Ca (4) Cervical Ca (1) CML (1) Colon Ca (1) Endometrial Ca (1) Hodgkin’s (1) Laryngeal Ca Melanoma (2) NHL (4) NSCLC (5) Oropharyngeal Ca (1) Ovarian Ca (1) SCLC (1) SqCC of thymus (1) Stomach Ca (1)

Ca, carcinoma; CML, chronic myeloid leukemia; FUO, fever of unknown origin; GCA, giant cell arteritis; HES, hypereosinophilic syndrome; NHL, non-Hodgkin’s lymphoma; NSCLC, non small cell lung carcinoma; PCNS, primary CNS vasculitis; PsO, psoriasis; RA, rheumatoid arthritis; SCLC, small cell lung carcinoma; SQCC, squamous cell carcinoma; TAK, Takayasu arteritis.

#### Indications for the performance of PET/CT

In patients who had a known history of vasculitis, PET/CT imaging was performed to assess disease activity and to demarcate the extent of vessel involvement. In patients with a history of malignancy, studies were performed for monitoring purposes. Seven of the patients in the cohort underwent PET/CT due to the presence of FUO and elevated inflammatory markers. Three patients with fevers and elevated inflammatory markers underwent PET/CT due to a strong suspicion for infection. Of them, two had undergone bioprosthetic aortic valve replacement with a suspicion of endocarditis and one had blood cultures positive for salmonella. In the patient with HES and in the patient with RA and PsO, a PET/CT was performed due to unexplained persistently elevated inflammatory markers.

### PET/CT findings

#### Vessel involvement

- Increased uptake along the aorta
  Of the 57 PET/CT studies evaluated, 39 (68%) demonstrated increased uptake in the aorta. Of these, 23 (59%) showed involvement of the thoracic aorta: 6 (26%) of which involved the arch alone, 4 (17%) with exclusive involvement of the ascending aorta, and 13 (57%) with involvement of multiple regions of the thoracic aorta. In 21 (53%) of the 39 studies with aortitis, involvement of the abdominal aorta was revealed. Five (23%) of these 21 also demonstrated increased uptake along the thoracic aorta. Six (29%) of the 21 showed aortic involvement extending into the bifurcation of the iliac arteries (Table 2).

**Table 2:** Arterial involvement according to clinical background.

Clinical background	Pattern of vessel involvement on PET	Areas of vessel enhancement on PET	Increased arterial wall thickness on CT (+ /−)	Extravascular inflammatory soft tissue features: pulmonary opacities
FUO, weakness, elevated inflammatory markers	Solitary	Arch	−	
	Multiple	Abdominal and thoracic aorta	+	
	Multiple	Abdominal aorta, b/l brachial aa, b/l subclavian aa, b/l vertebral aa, and b/l iliac aa	−	
	Multiple	b/l iliac aa, b/l subclavian aa, b/l brachial aa	+	
	Multiple	Abdominal and thoracic aorta, b/l brachial arteries	+	
	Solitary	Thoracic aorta	−	
TAK	Solitary	Arch	−	
	Solitary	Infundibulum and pulmonary trunk	+	LLL opacities
	n/a	No increased uptake	−	b/l opacities
		No increased uptake		RLL opacity
		No increased uptake		b/l opacities
		No increased uptake		
		No increased uptake		
	Multiple	Abdominal aorta and L renal a	−	
	Multiple	Ascending and descending aorta, L brachiocephalic a, L carotid a, L subclavian a	−	
	Multiple	Ascending and abdominal aorta	−	
GCA	Multiple	Abdominal aorta, b/l brachial aa, b/l subclavian aa, and b/l carotid aa	−	
	n/a	No increased uptake	−	
		No increased uptake	−	
GPA	Multiple	Abdominal aorta and arch	+	
	Solitary	Arch	+	
	n/a	No increased uptake	−	
HES	Multiple	Arch, ascending and descending aorta, L brachiocephalic a, L carotid a, and L laryngeal a	−	
RA and PsO	Multiple	Abdominal aorta and L iliac artery	−	RLL, RUL opacities
Behçet's disease	Multiple	Aortic bifurcation, b/l iliac aa	+	
PCNSV	Multiple	Common carotid a and R carotid a	+	
IgG4 related disease	Multiple	Main pulmonary r, R pulmonary a, L pulmonary a	+	RUL, bibasilar opacities
Infectious aortitis	Solitary	SMA	+	b/l opacities
			+	
		Root	−	
Mycotic aneurysm	Multiple	Root, ascending and abdominal aorta	+	
Adrenal tumor	Solitary	Abdominal, descending aorta	+	
Breast Ca	Solitary	Arch	+	
		Ascending aorta	−	RUL opacity
		Thoracic aorta	−	
		Abdominal aorta	−	
Cervical Ca	Solitary	Thoracic and abdominal aorta	−	
CML	Multiple	Abdominal aortal, b/l iliac aa	−	
Colon Ca	Solitary	Thoracic aorta	−	RUL opacity
Endometrial Ca	Solitary	L common carotid a	+	
Hodgkin’s	Solitary	Ascending aorta	−	
Laryngeal Ca	Solitary	L subclavian a	+	
Melanoma	Solitary	R common carotid a	+	
	Multiple	Ascending and descending aorta	−	LLL opacity
NHL	Solitary	Thoracic aorta	−	RML, RLL opacities
		Abdominal aorta	−	
	Multiple	Abdominal aorta	−	
		b/l carotid aa	−	
NSCLC	Solitar	Abdominal aorta	+	
		Arch and ascending aorta	+	
	Multiple	Ascending and descending aorta	−	
		Arch, L brachiocephalic v	+	RUL opacity
		Abdominal aorta, b/l iliac aa	+	
Oropharyngeal Ca	Multiple	b/l brachial aa	−	
Ovarian Ca	Multiple	Abdominal aorta, b/l iliac aa	+	
SCLC	Solitary	L subclavian v	+	
SqCC of thymus	Solitary	Ascending aorta	−	
Stomach Ca	Solitary	L carotid a	+	

a, artery; aa, arteries; b/l, bilateral; Ca, carcinoma; CML, chronic myeloid leukemia; FUO, fevers of unknown origin; GCA; giant cell arteritis; GGO, ground glass opacities; GPA, granulomatosis with polyangiitis; HES, hypereosinophilic syndrome; l, left; NHL, non-Hodgkin’s lymphoma; n/a, non-applicable; NSCLC, non small cell lung carcinoma; PCNSV, primary central nervous system vasculitis; PsO, psoriasis; r, right; RA, rheumatoid arthritis; RLL, right lower lobe; RML, right middle lobe; RUL, right upper lobe; SCLC, small cell lung carcinoma; SMA, superior mesenteric artery; SQCC, squamous cell carcinoma; TAK, Takayasu arteritis.
- Increased uptake along other large vessels
  Of the 57 PET/CT studies reviewed, 25 (44%) demonstrated increased tracer uptake in other large vessels. Of the 25, thirteen (52%) showed enhanced uptake in these vessels in addition to increased uptake in the aorta. Twelve (48%) patients had increased uptake in large vessels without evidence of inflammation in the aorta.
- Absence of vessel involvement
  Eight PET/CT results revealed an absence of increased tracer uptake in the vessels. These scans were performed on patients with known histories of vasculitis. Five of these scans were performed on patients with a known history of Takayasu arteritis who had been treated with high doses of steroids. One of them had also received azathioprine followed by infliximab. Two of the patients had a history of GCA and had been treated with high dose steroids. One patient been diagnosed with GPA 4 years prior to the PET/CT scan, had a consistent biopsy, and was subsequently treated with immunosuppressive treatment.
- Vessel involvement in the absence of malignancy, according to clinical background.
  In 6 of the patients who presented with FUO and elevated inflammatory markers, PET/CT showed evidence of aortitis. The scans of 3 of these patients also revealed increased uptake along other large vessels. In one of the patients who underwent PET/CT for FUO and elevated inflammatory markers, the scan revealed aortitis in the presence of mycotic aortic aneurysm. Among the 3 who underwent PET/CT for suspicion of infectious aortitis, imaging illustrated increased uptake in the superior mesenteric artery in 2 and in the aortic root in one.
  Of 10 patients who were treated for Takayasu arteritis, 4 showed evidence of aortitis on PET/CT scans; one of them also showed vasculitis in other large vessels. One patient's scan illustrated vasculitis in the infundibulum and pulmonary trunk alone. For 5 patients, no evidence of vasculitis was found. Of the 3 patients who had been treated for GCA, 1 had increased uptake along the abdominal aorta and along the great vessel superior, and inferior to the aorta bilaterally (Fig. 1). PET/CT scans of the remaining 2 patients did not show signs of active vasculitis.

**Figure 1:**
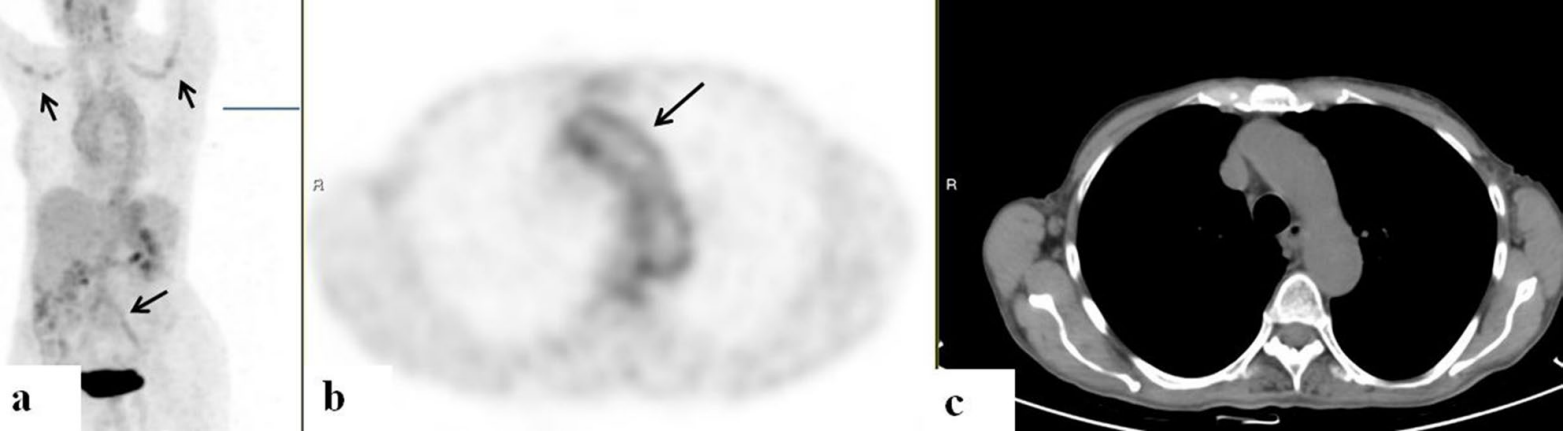
FDG-PET/CT: maximum intensity projection (MIP) (**a**) a representative PET (**b**) and CT (**c**) axial slices. A 75-year-old woman with giant cell arteritis. PET demonstrates increased uptake (arrows) along the vessel walls of the aorta, subclavian and common iliac arteries.
  Of 3 patients with a history of treated GPA, PET/CT studies revealed aortitis in 2. The PET/CT scans obtained from patients with HES, RA, PsO, Behçet's, and PCNSV demonstrated evidence of vasculitis in at least 2 large vessels. The scan from a patient with IgG4 disease displayed increased uptake in the large pulmonary arteries (Fig. 2).

**Figure 2:**
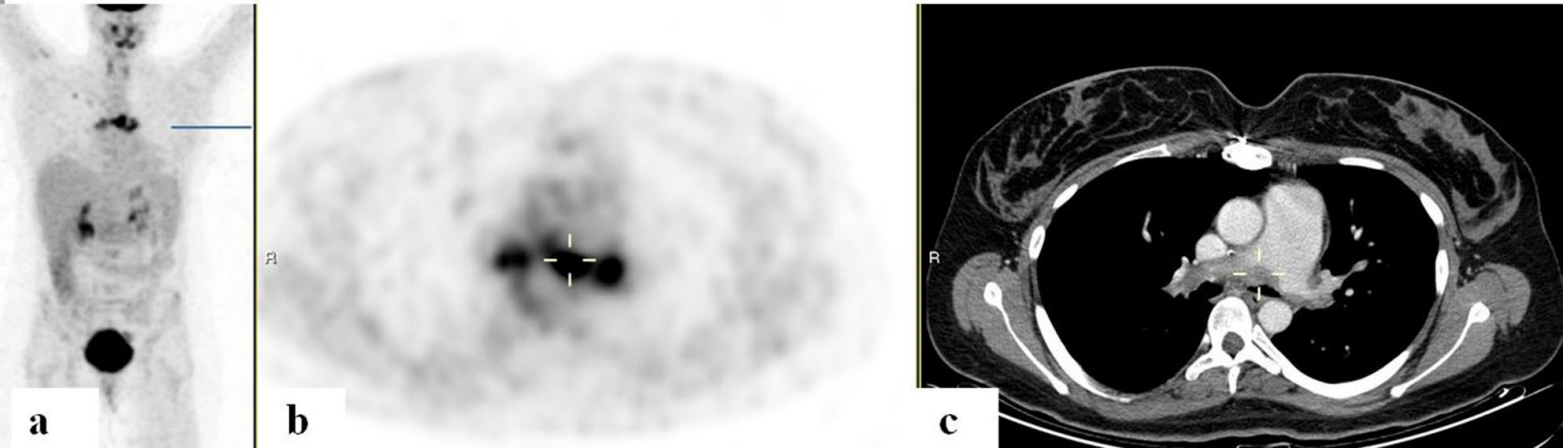
FDG-PET/CT: FDG-PET/CT: maximum intensity projection (MIP) (**a**) a representative PET (**b**) and CT (**c**) axial slices. A 31-year-old woman with IgG4-related disease with biopsy proven pulmonary arteritis. PET demonstrated increased uptake along the main pulmonary artery walls (cursers) with corresponding filler defects adjacent to the internal walls of the vessels on CT.
- Vessel involvement in patients with a history of malignancy.
  In the patients with a history of malignancy, areas of enhanced uptake on PET/CT tended to be more restricted than in patients without a history of malignancy. Among 20 patients with a history of solid malignancy, 6 (30%) showed enhanced tracer uptake in the thoracic aorta, 3 (15%) in the abdominal aorta, and 3 (15%) in both the thoracic and abdominal aorta. The PET/CT scans of one of these patients also involved the left brachiocephalic vein, while the scans of two others also involved bilateral iliac arteries. Six (30%) patients showed enhanced arterial wall uptake in the absence of aortic involvement. Areas of increased uptake included the common carotid artery in two patients, the left carotid artery, the left subclavian vein, the left subclavian artery, and bilateral brachial arteries.
  In the 6 patients with hematologic malignancies, two showed enhanced uptake in the thoracic aorta and three in the abdominal aorta. None of these patients showed enhanced uptake in any additional vessels. One patient's PET/CT scan showed enhanced uptake in the bilateral carotid arteries in the absence of aortic involvement.

### Wall thickening along the aorta and large vessels

Twenty-one (37%) of the PET/CT studies revealed thickening of the walls of the aorta. Of these, three (14%) illustrated increased tracer uptake in the thoracic aorta, 5 (24%) in the abdominal aorta, and 4 (19%) in areas of both the thoracic and abdominal aorta. Nine (16%) of the total studies demonstrated only increased uptake of the large vessels though not of the aorta itself.

### The presence of aortic aneurysm

**Figure 3:**
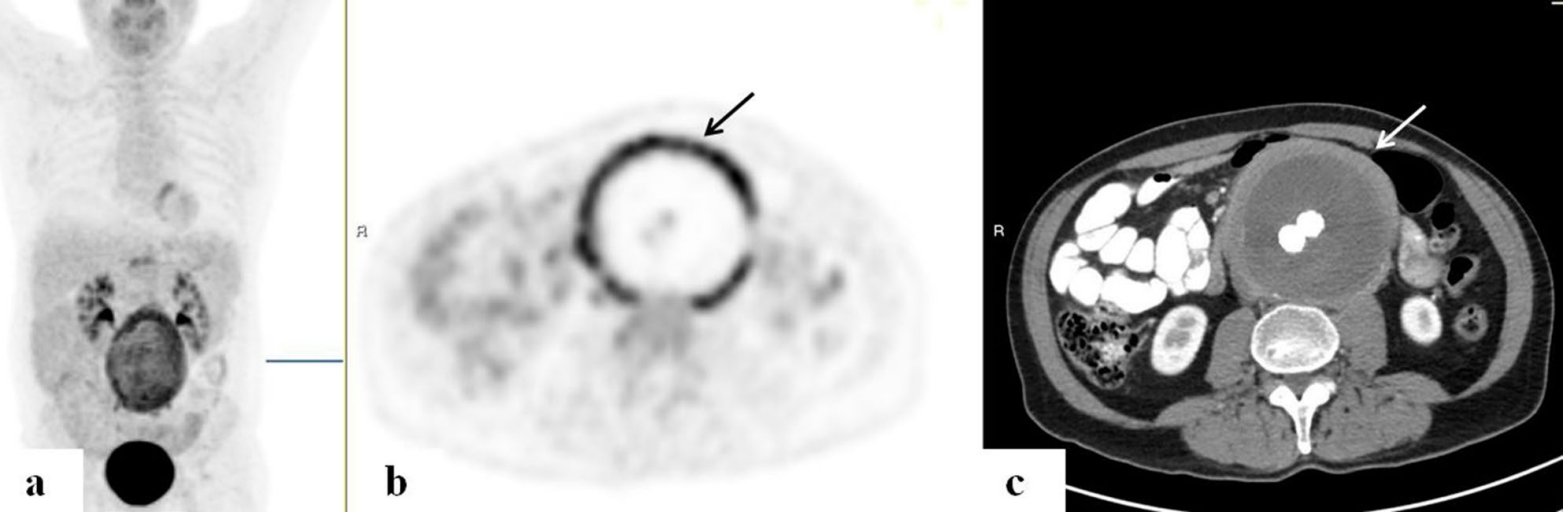
FDG-PET/CT: maximum intensity projection (MIP) (**a**) a representative PET (**b**) and CT (**c**) axial slices. A 65-year-old man with mycotic aneurism of the abdominal aorta. PET demonstrates high intensity of increased uptake (arrows) along the markedly thickened wall of the dilated abdominal aorta following repair.

Evidence of an abdominal aneurysm with enhanced uptake in that area, and extending into the bifurcation and along bilateral iliac arteries, was detected in a patient who was being monitored for a history of lung cancer. In another patient who was evaluated for FUO and very elevated inflammatory markers with a C-reactive protein (CRP) as high as 230 mg/dl (normal range 0–5), a mycotic aneurysm in the abdominal aorta was detected on the PET/CT. He was treated accordingly with surgical repair (Fig. 3).

### The presence of arterial thrombus

**Figure 4:**
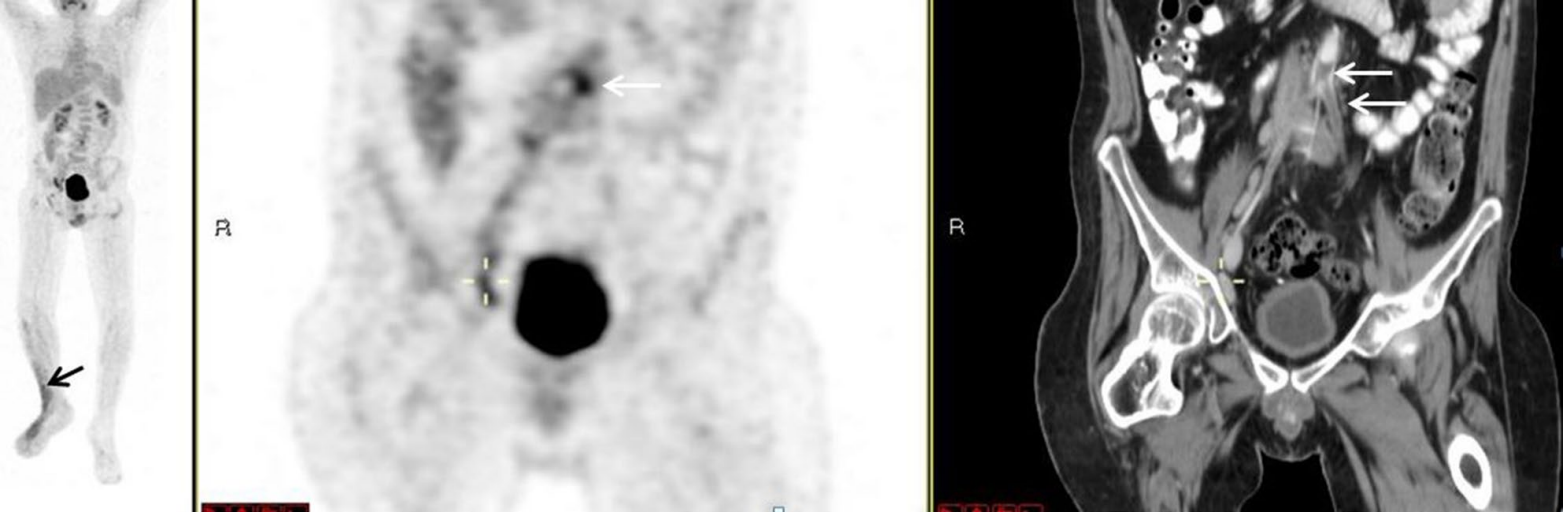
FDG-PET/CT: FDG-PET/CT: maximum intensity projection (MIP) (**a**) a representative PET (**b**) and CT (**c**) coronal slices. A 37-year-old man with Behçet's disease. PET demonstrated increased uptake along the filling defect in the aorta at the bifurcation extending to the iliac arteries (white arrows, **b**, **c**) and increased uptake in the soft tissues of the Rt leg (black arrow, **a**).

Evidence of a 2 cm thrombus along the superior mesenteric artery was demonstrated in the PET/CT of a patient who had salmonella bacteremia. Heightened tracer uptake presented in that area, with an SUVmax of 2.3. In the patient with Behçet's, PET/CT highlighted a filling defect along the bifurcation of the iliac arteries. Enhancement was increased in the regions both proximally and distally, which extended into the bilateral iliac arteries (Fig. 4).

### The presence of synovitis

Of the ^18^F-FDG PET/CT scans performed, 4 (7%) revealed evidence of synovitis. One of these scans was performed in a patient with a history of GCA complicated by active polymyalgia rheumatica (PMR), with a persistently elevated CRP level despite treatment with high dose steroids. PET/CT did not show evidence of vasculitis. However, it did show increased uptake along the shoulder and pelvic girdles.

Another PET/CT scan showed increased uptake along the bilateral shoulders in a patient with a prior history of metastatic breast cancer, with evidence of vasculitis along the ascending aorta. In one woman with a history of endometrial cancer and vasculitis of the left common carotid artery, the PET/CT revealed increased uptake along the left shoulder. Lastly, one patient with a prior history of melanoma with PET/CT evidence of vasculitis along the ascending and descending aorta, also demonstrated increased ^18^F-FDG uptake, which was along the right knee.

## Discussion

We reviewed the PET/CT scans performed in our medical center for the evaluation of vasculitis over a 10-year period. We found evidence of LVV in 49 studies. The 8 scans that did not reveal active evidence of vascular inflammation were performed in patients who had received immunosuppressive treatment: 2 with GCA, 5 with Takayasu arteritis, and 1 with GPA. Seven patients were evaluated by PET/CT due to FUO and elevated inflammatory markers; 2 reported weakness and diffuse body aches. The aorta was enhanced in all of their scans and 2 had inflammation in other large vessels. Two of the 6 patients met the criteria for a final diagnosis of GCA while 1 patient met the criteria for a diagnosis of Takayasu arteritis.

Almost one-third of the PET/CT scans were performed in patients who had a known diagnosis of vasculitis. In some of the patients with a background diagnosis of GCA and Takayasu arteritis, the PET/CT served as a useful tool for illustrating increased uptake and demonstrating vasculitis. While the challenge of distinguishing active inflammation from vascular remodeling remains, the findings on PET/CT appeared to correlate with other features of active disease.

One of our patients with previously treated GCA and half of those presenting with FUO who were eventually diagnosed with GCA showed evidence of extratemporal LVV on PET/CT. These numbers collaborate reports of extratemporal involvement in GCA in close to half of the scans performed^8^. Furthermore, in one third of our patients who underwent PET/CT due to a suspicion of vasculitis in the setting of FUO, the scans showed evidence of LVV. This supports the utility of these studies in distinguishing this entity. The use of PET/CT to evaluate FUO has grown in popularity due to its sensitivity and specificity in detecting metabolic changes seen even prior to clinical manifestations, and because LVV is a common explanation for FUO. LVV accounts for close to one fifth of the cases of FUO in patients over age 50 years, most commonly is GCA among the elderly^25,26^.

Of the 3 patients with partially treated GPA, two showed increased large vessel uptake. The increased uptake was present in the aortic arch in one of them and along the arch, abdominal aorta, and bilateral iliac arteries in the other. This is consistent with findings in the literature that have shown large vessel involvement in GPA^27^. Moreover, FDG-PET/CT enables the evaluation of vessel wall inflammation in cardiac, sinonasal, lung, and kidney vascular beds in ANCA-associated vasculitis^28^.

Our study also revealed evidence of aortitis and large vessel vasculitis in patients with Behçet's, IgG4 related disease, RA, and PsO. Such findings are consistent with other reports in the medical literature. Abnormal uptake on PET/CT has been demonstrated in the aorta, carotid artery, and superior mesenteric artery in persons with Behçet's disease^29^. PET/CT is also useful in assessing for vasculitis in IgG4-related disease, in which aortitis, often with adjacent aneurysm, has been reported in up to 36% of cases. These generally result from perivascular tumefactive lesions^30^. In addition, PET/CT can distinguish cardiovascular features of IgG4-related disease including aortitis, periaortitis, arteritis, periarteritis, atherosclerosis, and pericarditis^31^. Moreover, areas of aortic inflammation may be apparent on PET/CT scans in patients with PsO and RA, even after adjustment for cardiovascular risk factors^32^.

In our patient in whom HES was previously diagnosed, increased uptake was seen in the thoracic aorta and in 3 large neighboring vessels. In the patient with a diagnosis of PCVNS, PET/CT demonstrated vasculitis of bilateral carotid arteries. The ^18^F-FDG PET results of these two patients suggest that an alternative diagnosis of systemic vasculitis should be considered.

Almost half the PET/CT studies of patients who were being monitored for recurrent malignancy showed enhanced uptake in the large vessels. Only one fifth of them were still receiving active treatment. These scans tended to show more restricted enhancement, either in the aorta or in one of the large proximal branches. One quarter of the patients who underwent PET/CT due to monitoring for recurrent malignancy, all with a history of solid tumor, exhibited evidence of inflammation in the aorta, and in one or two additional adjacent vessels.

The findings of increased arterial wall uptake in patients with malignancy highlights that the appearance of large vessel inflammation on PET/CT should prompt a thorough evaluation to exclude the presence of malignancy. LVV itself does not appear to be associated with increased risk of malignancy. Nonetheless, arterial wall inflammation in the absence of clinical features of vasculitis presents in a sizable subset of cancer patients. The exact pathogenesis remains unclear though may be related to an altered immunologic response to cancerous (neo)antigens that share homology with vascular antigens^33^. Moreover, the use of both granulocyte colony stimulating factor (G-CSF) therapy and taxane chemotherapy have been associated with the emergence of LVV^34^. Prior groups have postulated that chemotherapy may interfere with the clearance of immune complexes and this may result in the involvement of neutrophil receptors along the vasa vasorum of the vessel walls. Additionally, the proliferation of neutrophils caused by G-CSF may lead to a cascade of uncontrolled vascular inflammation^35^. Accordingly, incidental identification of LVV in cancer patients warrants careful review of all the prior medications received.

For a small proportion of our patients, PET/CT was useful in highlighting sites of synovial inflammation. In particular, the increased uptake along the shoulder and pelvic girdles in a patient with quiescent GCA confirmed the suspicion of coexistent active PMR. This value of PET/CT was previously demonstrated among 16 patients with definitive PMR, of whom enhanced ^18^F-FDG uptake presented in the glenohumeral and sternoclavicular joints in 88%, and in the greater trochanters in 81%^36^.

Our study relied on the use of PET/CT. Magnetic resonance imaging (MRI)/angiography may also offer a wide range of vascular evaluations. MRI can reliably evaluate the small temporal arteries and assess findings of both vascular inflammation and damage with a more accurate estimation of the lumen patency than obtained from PET/CT^37,38^. While each modality has its advantages, the combination of PET and CT can evaluate the whole body and assess for increased metabolism, while also accurately delineating vessel diameter^39^.

There are several limitations to our study including its retrospective nature, small sample size, and its setting in a single institution. Additionally, we included a heterogeneous group of patients with a variety of background diseases and we did not have pathological correlation of the vasculitis findings seen on PET/CT. Moreover, in the absence of a healthy control group, it is difficult to conclusively state that the abnormalities detected truly reflect vasculitis rather than atherosclerotic lesions, non-inflammatory smooth muscle metabolic activity or proliferation, or other non-specific changes. Nonetheless, the careful characterization of PET/CT findings in this study, together with the evaluation of available clinical information and laboratory work strongly suggest that the vascular changes identified on the PET/CT scans represented LVV in a spectrum of patients. Our study also highlighted the caution that must be taken in interpreting PET/CT findings in patients with malignancy.

The use of PET/CT to diagnose LVV remains with drawbacks. With the initiation of glucocorticoids, which is the mainstay of early therapy in non-infectious vasculitis, the accuracy of PET/CT drops dramatically^40^. Moreover, higher FDG uptake may be seen in aging vessels as a result of changes in metabolic activity, vessel wall remodeling and atherosclerosis^41^. Accordingly, over the last twenty years, scientists have worked on developing macrophage targeted tracers^42^. As the field continues to evolve, we are likely to see PET/CT adapt new radiotracers with target specific biomarkers which, in the case of LVV, will highlight specific subsets of macrophages^43^. Moreover, as the use of theranostic radiopharmaceuticals advances, nuclear medicine may also provide a way to offer precision treatment of LVV^44^.

## Conclusion

Overall, our findings illustrate the ability of PET/CT to outline large vessel vasculitis. This modality enables the assessment of the entire vasculature for sites of inflammation. In our study, it confirmed the presence of extracranial inflammation in GCA. Moreover, it detected active inflammation among patients with Takayasu arteritis and GCA who had previously been treated with immunosuppression. PET/CT also demonstrated the presence of large vessel inflammation in diverse autoimmune diseases including GPA, IgG4 disease, RA, and Behçet's disease. Finally, PET/CT showed restricted large vessel wall inflammation in several patients who were being monitored for the recurrence of malignancy.
